# The crisis of public trust and legitimacy in public health: being successful in a world in turmoil

**DOI:** 10.1093/pubmed/fdaf099

**Published:** 2025-11-18

**Authors:** Bruce Jennings, Lawrence O Gostin

**Affiliations:** Department of Health Policy and the Center for Biomedical Ethics and Society, Vanderbilt University, 2525 West End Ave., Suite 400, Nashville, Tennessee, 37203, USA; Department of Health Policy and Core Faculty of the Center for Biomedical Ethics and Society, Vanderbilt University, 2525 West End Ave. Suite 400, Nashville, TN 37203 USA; Global Health Law, Georgetown University, 37th and O Streets, NW, Washington, DC 20057, USA

Public health is at a moment of crisis in trust. Emerging from the COVID pandemic, the world seems in turmoil. Science, and truth itself, is politically and socially contested. Social media and artificial intelligence have amplified falsehoods. And the public is no longer willing to accept the authority and legitimacy of public health experts. The field of public health has found itself unable to successfully operate in this new world in turmoil.

To be effective public health needs a cultural and conceptual context in which to function. Public health’s appeal to people to adhere to healthy behaviors is no longer accepted because the basic groundwork of value, obligation, and motivation have lost their force and appeal for many. We need to approach the problem of trust and authority in a way that can appeal to social and political values in new ways. The main point is that the crisis facing public health now is a civic and cultural crisis. To function more effectively, the field needs to revitalize a commitment to civic values, community welfare, and mutuality as a way of life.

## The great privatization

The impact of the early days of the Trump administration in the United States has been highly disruptive and distracting—not only for Americans but for people everywhere. Seismic disruptions have occurred in virtually every realm, particularly deep cuts to scientific research and public health, evisceration of foreign health assistance, and anti-science and anti-vaccination crusades at the very highest levels of government. And there are other assaults to the body politic, such as intrusive collection of personal and sensitive information in areas ranging from health to tax and immigration. Civil servants, including in health and science, have been fired or forced into retirement.

All this has been little more than a massive privatization maneuver aimed in the health sector at high-functioning and very well-established institutions, such as the National Institutes of Health, the Centers for Disease Control and Prevention, and the Environmental Protection Agency. It deeply impacts a wide range of people who form the infrastructure of medical and health expertise and creative innovation.

This lost expertise will not easily be recovered or replaced. With cascading crises on the horizon (e.g. climate change, mass migrations, and pandemic risks), one could hardly find a worse time to be disassembling the leaders in science, public health, and health administration. All of this and more will play out against the backdrop of aging demographics, increased vulnerabilities, and rising inequalities. Societies will struggle with the systematic and institutional challenges of a planned and humanely administered response to social ills.

In sum, the field and practice of public health is at a point of deep crisis in trust and legitimacy. We note this as we intend to offer some concerns and aspirations for the future of public health and health policy. Soon virtually all the helping and care-giving professions will be drawn under the umbrella of “public health.” The current leadership of public health will see the magnitude of the problems it faces due to the breakdown (both financial and otherwise) of “privatized health.”

## The enigma of the public and its health

Public trust usually goes hand in hand with a cluster of norms and attitudes toward trust, trustworthiness, confidence in leadership, impartiality, and respect for scientific expertise and public health guidance. This is an everyday, basic task, and it switches into high gear in times of crisis or emergency situations. And yet, opinion polls show declining belief and almost automatic dismissal of information coming from any source identified with public health officials. The view seems to stem from a feeling of non-recognition or wrong recognition by arrogant experts and elites who do not respect ordinary working people.

Moreover, the era of public health’s and medicine’s unquestioned authority is over. The contemporary generation embraces a simple and essential truth—there are values that are just as important to individuals and to society as public health. These values include education, economic prosperity, personal rights and freedoms of choice, the safety of their children, and equitable access to needed preventive and lifesaving health care.

This was a large part of the message heard during and after COVID 19. The importance of this changing worldview must not be ignored. Where does this conflict and politization come from? A complex question to be sure, but it is only one facet of the challenges facing public health in times of turmoil, which are really times of “increasing” turmoil. Turmoil is foreseen as a chronic condition, not an episodic one.

A hallmark of the population perspective on health today is on the broad notion of the “social determinations of health.” Applied to ourselves in the field, we suggest that in the coming months we convene to ask “What are the social determinants of our own field of public health? What capabilities and institutional patterns of behavior must be in good working order if the field and profession of public health is to recover from its current malaise? We will address the normative, but also profoundly motivational and implicit questions of the current health of public health as such and its prognosis?

## Recognizing and relating: toward a more civic public health

To be effective, public health needs a “cultural and conceptual context in which to function.” Many of us thought that the basic values of public health and the political commitment to the field would remain a bedrock of modern society. Now it seems that we were complacent, possibly arrogant. It is not just the polite framing of “distrust” but rather monumental struggles between public health and the body politic. Public officials were elected precisely because they were perceived as warriors against the scientific elites. The mutual antipathy may well have become structural rather than merely transitory. If so, that will make the conversations we have today very difficult. And the solutions will take hard work.

Norman Daniels, a highly respected scholar in public health said, “Justice is good for your health.” He meant that everyone has a right to be treated with equal respect and dignity as a person whose life matters ethically. If public health, as we know it, is to bounce back and right its own boat, then public health practices and policies will need embrace values that see the wants and needs of the public. And that means public health values will have to permit and encourage certain types of freedom and personal autonomy. This seems at odds with the primary principle of public health, which is the population perspective. We strive for the common good, and in doing so we have long believed that individuals may have to forgo some of their personal freedoms.

Our suggestion is that some of the difficulty experienced by those practicing public health arises from the ideologicalworld views predominant today that prize personal and economic freedoms more than population health. For many, this takes away the satisfaction found in the work of the public health profession. Even before the COVID-19 pandemic, public health professionals did not fully appreciate the values and priorities being voiced by many parts of society. This was not entirely a political or ideological matter.

One flash point remains the attitude of many parents that seem troubled by vaccination. It certainly is a clash between secular and religious values but goes well beyond that dichotomy. There are many parents who are secular and not particularly religious but who reject vaccination for what many in public health consider spurious reasons. Finally, there is considerable mistrust of and antipathy toward the pharmaceutical industry, who are perceived to drive uptake of their products like vaccines. Another group at war with public health are libertarians who refuse to social distance or wear a face mask on the grounds of freedom of choice and action. And finally many in society, well beyond ideological libertarians, who bristled at public health advice to shutter business activities and to deprive school children of cognitive and social development.

We advance the notion that public health as a profession cannot fight these disputes alone, nor should it have to. The problem is fundamentally cultural and political, but we do not mean by that who has the most power. When we say that the problem is political, we mean that social, economic, and even cultural or religious disputes should be understood as fellow citizens asking us to respect their values and choices. This requires self-examination, compromise, and reconciliation. People need right recognition so that they can see that their views and interests are honored, valued, and respected even if your interest or position in a dispute does not prevail. And people need right relationships because having these civic friendships give one the solidarity and security that are needed to pursue a community dispute in which the stakes are very high. Inducement to join in with this reconciliation pivots on the fact that by doing so you are not only recognizing your rights, but you are also recognizing that there is something at work in these deliberative acts and conversations that is larger than anyone’s private interests. This requires a new form of civic thinking that will be slow to form, hence civic democracy exemplifies patience, perhaps the most underrated political virtue.

To bring about the institutional and behavioral renewal of the sort sketched here, it is necessary for public health today to see health as genuinely “public,” as something that involves us all, as a common good, not as a commodity we pay for and consume. The quality of our collective and individual health depends upon an intricate web of cooperation and interdependence. But, just as large areas of our society are food deserts where people lack access to healthy diets, so too large areas are civic deserts with virtually no organized and ongoing settings for deliberation about our common lives together.

Of course, the analogy is not perfect. Food deserts tend to exist in low income and marginalized areas since nutritious foods are privately distributed as a market commodity. (The vital National School Lunch Program and Meals on Wheels nutritional programs for seniors are exceptions that prove the rule.) Civic deserts are created more by cultural and political attitudes of privatization and exclusion rather than by inequalities of wealth. Indeed, where food deserts exist, civic communities often combat that deprivation and other forms of environmental and health injustice. We shall deepen the tragedy that the COVID-19 pandemic has caused if we fail to find the leadership and political will to revitalize our democracies by enhancing opportunities for civic practice and civic engagement that is ethically informed. We require nothing less than a new civic conscience for public health.

## Notes

**Bruce Jennings,** MA. Political Science and Philosophy, Adjunct Professor, Vanderbilt University

**Lawrence O. Gostin,** JD. Law is University Professor and Founding O'Neill Chair in Global Health Law, Georgetown University

